# Impact of extreme weather conditions on European crop production in 2018

**DOI:** 10.1098/rstb.2019.0510

**Published:** 2020-09-07

**Authors:** Damien Beillouin, Bernhard Schauberger, Ana Bastos, Phillipe Ciais, David Makowski

**Affiliations:** 1 CIRAD, UPR HortSys, 34398 Montpellier, France; 2 HortSys, University Montpellier, CIRAD, Montpellier, France; 3 Potsdam Institute for Climate Impact Research (PIK), 14473 Potsdam, Germany; 4 Laboratoire des Sciences du Climat et de l'Environnement, Institut Pierre-Simon Laplace (IPSL), 91191 Gif sur Yvette, France; 5 Ludwig-Maximilans-Universität Munich, Luisenstrasse 37, 80333 München, Germany; 6 INRAE, AgroParisTech, UMR 211 Agronomie, Université Paris-Saclay, 78850 Thiverval-Grignon, France; 7 CIRED - Centre international de recherche sur l'environnement et le développement, UMR 8568, Nogent-sur-Marne, France

**Keywords:** climate extremes, crop yields, drought, yield anomalies, wheat, random forest

## Abstract

Extreme weather increases the risk of large-scale crop failure. The mechanisms involved are complex and intertwined, hence undermining the identification of simple adaptation levers to help improve the resilience of agricultural production. Based on more than 82 000 yield data reported at the regional level in 17 European countries, we assess how climate affected the yields of nine crop species. Using machine learning models, we analyzed historical yield data since 1901 and then focus on 2018, which has experienced a multiplicity and a diversity of atypical extreme climatic conditions. Machine learning models explain up to 65% of historical yield anomalies. We find that both extremes in temperature and precipitation are associated with negative yield anomalies, but with varying impacts in different parts of Europe. In 2018, Northern and Eastern Europe experienced multiple and simultaneous crop failures—among the highest observed in recent decades. These yield losses were associated with extremely low rainfalls in combination with high temperatures between March and August 2018. However, the higher than usual yields recorded in Southern Europe—caused by favourable spring rainfall conditions—nearly offset the large decrease in Northern European crop production. Our results outline the importance of considering single and compound climate extremes to analyse the causes of yield losses in Europe. We found no clear upward or downward trend in the frequency of extreme yield losses for any of the considered crops between 1990 and 2018.

This article is part of the theme issue ‘Impacts of the 2018 severe drought and heatwave in Europe: from site to continental scale'.

## 1. Introduction

Interannual instability in agricultural production can threaten local and global food security [1]. The growing frequency or intensity of extreme weather events [2] may increase the risks of multiple simultaneous crop failure within regions or globally [3,4]. Quantifying yield loss anomalies at large spatial scales and understanding their climatic drivers is a prerequisite to assess vulnerabilities and design adaptation measures to increase the resilience of food systems [5,6]. Yet, the multiplicity of factors involved such as the nature, timing and intensity of extreme weather conditions, crop species and management complicates the prediction of yield losses [7–9]. Recent studies indicate that compound extremes need to be considered additionally to single climate extremes [10–12]. Process-based crop models incorporate crop growth mechanisms but have moderate ability to reproduce historical crop yield anomalies [13–15]. Statistical models offer alternative support for the attribution of climate impacts on crop yields (e.g. [8,9,16]). Attribution analyses can be performed at national scales, but spatial heterogeneities in yield, climate and soil conditions can be important. It is hence expected that statistical and machine learning models perform better when using yield and climate data at sub-national scales. Even during recent droughts and heat waves in Europe, it was shown that within the same country, some regions still experienced normal or even wetter conditions [12]*.* In 2018, Northern, Central and Eastern Europe faced unusual simultaneous extreme temperature and dry conditions from March to August, whereas several areas in Southwestern Europe were exposed to higher rainfalls. The multiplicity and diversity of atypical climatic conditions in 2018 make this year a particularly interesting case to better understand the impact of extreme climatic events on agricultural yields in Europe.

In this study, two complementary analyses are presented: (i) a characterization of influential climate drivers on European crop yield anomalies at district scale based on historical time series, and (ii) an exploration of the impacts of extreme weather conditions in 2018 on yield anomalies. We rely on yield data from more than 1400 sub-national geographical units called districts, representing 17 countries, for nine major annual crops: barley, maize, oats, oilseed rape, potatoes, triticale, rye, sugar-beet and wheat. Past yield anomalies in the main European production areas are compared with those that prevailed in 2018. Then, on the basis of results from machine learning models (random forest), we identified the critical climatic drivers that exhibit a strong association with extreme yield anomalies observed in different European regions in 2018. Our results provide a better understanding of the climatic conditions that can lead to severe yield losses in Europe.

## 2. Material and methods

### (a) Yield anomalies and climate data

Crop yield time series were collected from yield data reported at the regional level in 17 European countries (i.e. at Nomenclature of Territorial Units for Statistics from EUROSTAT (NUTS) 2 and 3—electronic supplementary material, figure S1). Across the 17 countries, 1435 geographical units (hereafter called districts) were included. The length of the time series differed among countries, with the earliest time series starting in 1901 for France (electronic supplementary material, figure S1). Nine crops were considered: six cereals including winter and spring types, two tuber crops and one oilseed crop (electronic supplementary material, figure S1). For maize, only grain maize was included in the study. Irrigated and rainfed yields were not systematically distinguished in the official data.

In each district, the normalized yield anomalies were estimated empirically considering the long-term increase of yield, related to technological improvements and possibly to rising CO_2_ and other environmental factors as follows:

2.1 $${\bar{a}}_{i,t}=\frac{\left(Y_{i,t}-\mu_{i,t}\right)}{\mu_{i,t}},$$

where ${\bar{a}}_{i,t}\;$is the normalized yield anomaly in the *i*th district at year *t,* $Y_{i,t}\;$is the observed yield and $\mu_{i,t}$ the expected yield. The expected yield (*μ*_*i,t*_) corresponds to the long-term yield estimated by a statistical fit to the historical data. We applied a locally weighted scatterplot smoothing (loess [17]) to calculate this long-term component of each time series at district level. For each crop and district, normalized yield anomalies were then expressed as a percentile of the long-term time series. To do so, three probability distributions were fitted (i.e. normal, Cauchy, logistic) to each time series, with the R package *fitdist* [18]. The distribution with the lowest Akaike information criterion (AIC) was chosen to calculate the percentiles corresponding to each value in the time series of normalized anomalies, including that corresponding to the year 2018.

Percentiles of 2018 yield anomalies were then mapped at the NUTS3 scale using the Eurostat R package [19]. When only NUTS2 data were available, the data were processed at this spatial scale. Extreme low (high) yields were defined as yields lower (higher) than the 10% (90%) percentiles. Cumulative areas with extreme low and high yields were computed year by year for each crop at the European scale, and for four regions being Northern, Southern, Eastern and Western Europe (see electronic supplementary material, figure S1).

We used climate data from the ERA5 atmospheric re-analysis running from 1 January 2000 to 31 August 2018 [20]*.* The ERA5 climate variables on hourly temporal resolution at 0.25 × 0.25-degree resolution (about 20 km) were aggregated to daily time steps for each district. Nine climate variables were selected as predictors of yield anomalies, as listed in electronic supplementary material, table S1. We chose to base our analysis on those ‘simple' climatic variables as in Vogel *et al.* [8,9], based on the evidence that no obvious relationship has been established between the level of complexity of climate indicators and their accuracy for predicting yield anomalies, including extreme ones [21,22]. The daily values were aggregated over three periods of two or three months, i.e. January–February (JF), March–April–May (MAM) and June–July–August (JJA).

### (b) Impact of climate on yield

We used Random Forest (RF, [23]) models to predict normalized crop yield anomalies as a function of the eight climate variables for the three periods of the year for all available years, and then compared the results to the anomaly predicted for the year 2018 alone. Random forest is a machine learning method, which uses an ensemble of decision trees and can be applied to regression and classification problems [23]. It includes several tuning parameters that need to be trained from data, in particular the number of trees, number of candidate inputs at each node, and minimum number of data (i.e. yield anomalies) in each final node. RF models were trained for 36 pairs of crop x region (nine crop types times four regions) separately. For each crop × region, RF were first trained using all available years and, then, using only the data available in 2018, leading to two different models. All available districts were used to train the models in both cases. We also studied the ability of the RF models trained with all years to predict specifically year 2018. During the training procedure, the tuning parameters of RF were optimized using a cross-validation with a validation set including 25% of the data (electronic supplementary material, table S2). The criterion maximized during the training procedure was the proportion of the normalized yield variance explained (*R*^2^). RF models were implemented using the *ranger* R package [24].

For each RF, we ranked the individual climate input drivers according to their relative importance for predicting normalized yield anomalies. Variable importance values were calculated based on a metric that captures the increase in mean squared error (MSE), calculated from out-of-sample predictions, after randomly permuting the values of the respective predictors. Variable importance values in the RF were computed using the R *vip* library [25].

The functional relationships between input climate drivers and yield anomalies were analyzed by plotting one- and two-dimensional partial dependence graphs. A partial dependence graph shows the marginal effect of one or two exogenous features (here, one or two climate inputs) on the outcome predicted by the RF, compared to averages over the values taken by the other inputs. These graphs allow visualizing the effect of the variables considered on the predicted normalized yield anomalies. Partial dependence plots were derived using the *pdp* R package [26]. Here, we drew a partial dependence plot for the two most important climate drivers identified for each crop and each region. The partial dependence plots were used to show how combinations of those two most important climatic drivers associate with yield anomalies in the RF models over all considered years, and to highlight their influence in the year 2018.

We tested the robustness of our results by analysing the impacts of three detrending methods of the yield-time series (locally weighted scatterplot smoothing, polynomial linear regression and cubic splines [27]) on the outputs of our RF models. We also assessed the effects of alternative values of the tuning parameters of RF (electronic supplementary material, figure S2), and of using a gradient boosting algorithm ([28]; implemented in R package xboost [29]) instead of RF. Finally, we compared the responses provided by the partial dependence plots to those provided by accumulated local effects (ALE; implemented in ALEPlot package [30]), i.e. a plot showing the effects on yield anomalies of local changes in climate inputs.

## 3. Results

### (a) Impacts of climate on European crop yields

Depending on the crop species and region, RF models explained between 0 and 65% of the variance (mean value across crops = 34%) of normalized yield anomalies all years included, based on out-of-bag cross-validation (electronic supplementary material, figure S3). The most accurate models across regions were obtained for winter wheat (mean *R*^2^ = 43%) and sugar beet (mean *R*^2^ = 42%—electronic supplementary material, figure S3). The other crops all have more than 25% of the yield anomalies variability explained. The predictive quality of the RF models was relatively high for the Northern and Western European regions (mean *R*^2^ = 46% in both regions), intermediate for Eastern Europe (mean *R*^2^ = 34%), and low for Southern Europe (mean *R*^2^ = 15%).

**Figure 1.**
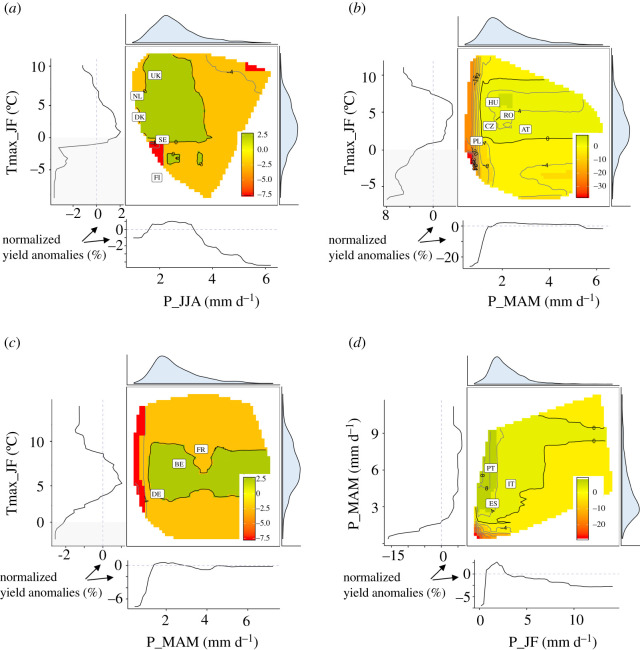
Effects of main climatic drivers on normalized winter wheat yield anomalies for Northern (*a*), Eastern (*b*), Western (*c*) and Southern Europe (*d*). The central panel for each region shows the combined effect of the two most important drivers on the normalized yield anomalies (in percent; different colour scale between regions). Only common values of climatic variables experienced by the crops are coloured (Finland experienced unusual combinations of P_JJA and Tmax_JF in 2018). Individual effects of the two drivers are presented at the left and bottom margins of each panel. The density curves at the top and the right of the central plot depict the distribution of the variables. The mean values observed in 2018 over all districts for a country are indicated by the country initials in the white boxes. (Online version in colour.)

**Figure 2.**
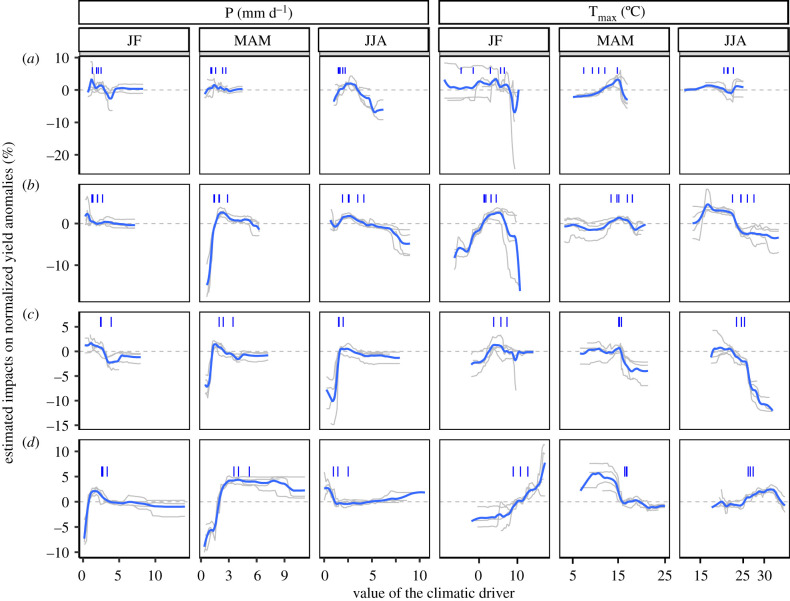
Estimated impacts of main climate drivers on cereal (i.e. barley, oats, triticale, rye, wheat) yield anomalies in four European regions: Northern Europe (*a*), Eastern Europe (*b*), Western Europe (*c*) and Southern Europe (*d*). Grey curves correspond to the effects of each cereal crop estimated independently by random forests based on historical times series. Only the six main drivers are presented in this plot (P, rainfall; T_max_, maximum temperature; JF, January–February; MAM, March–April–May; JJA, June–July–August). Blue curves correspond to a loess fit over all crops. Blue segments correspond to the experienced values of climate drivers in 2018 for each country within each region. (Online version in colour.)

No single climatic variable explained a large fraction of yield anomalies across crop species and regions. For example, rainfall or maximum temperature ranged between 10 and 20% of variance explained (electronic supplementary material, figure S4). Nonetheless, similarities of influential variables were noticeable among regions. In Northern Europe, large negative yield anomalies were mostly associated with sub-optimal temperatures in spring (mean value of Tmax_MAM less than 11°C or greater than 16°C) or with high rainfall in summer (P_JJA greater than 3 mm day^−1^; figures 1 and 2). Temperatures in January and February (Tmax_JF) played an important role in explaining wheat yield anomalies (figure 1), but its effects varied for other crops species. In Eastern Europe, yield losses were also associated with high summer rainfall (mean P_JJA greater than 5 mm day^−1^) and with high summer temperature (mean Tmax_JJA greater than 24°C). Rainfall deficit in spring (mean P_MAM less than 1.5 mm) or cold temperature in winter (mean Tmax_JF less than 0°C) were also found to lead to low yields. In Western Europe, low spring and summer rainfall (mean P_MAM less than 1 mm day^−1^; mean P_JJA less than 2 mm day^−1^) and high temperatures in the second part of the crop cycles (mean Tmax_MAM greater than 16°C; mean Tmax_JJA greater than 23°C) generally led to high negative yield anomalies. Cereal yields in Southern Europe were generally negatively impacted by low winter and spring rainfall (P_JF less than 1 mm day^−1^; P_MAM <∼ 2.5 mm day^−1^; figure 2) or by high temperatures in spring (mean Tmax_MAM greater than 17°C). On the contrary, high temperature in winter tended to increase yields (mean T_JF greater than 12°C in JF). The optimal summer temperatures (Tmax_JJA) seemed to range from 26 to 32°C.

The ability of the RF models calibrated on historical periods to predict specifically the normalized yield anomalies of 2018 varied between crops and regions (electronic supplementary material, figure S3). The *R*^2^ ranged between 0 and 80%, with relatively higher value in Northern (mean *R*^2^ = 50%) and Western Europe (mean *R*^2^ = 36%) but the RF had low predictability for Southern Europe (mean *R*^2^ = 13%). In Eastern Europe, the RF models calibrated on all years showed a large decrease in their performance to predict 2018. A large decrease of explained variance for the 2018 anomalies compared to all years was also observed for other specific combinations of crops and regions, e.g. rye in Western Europe or sugar-beet in Southern Europe. The ability of the models to predict 2018's yield anomalies were higher when the RF models were trained only with 2018 data (mean *R*^2^ = 0.36 versus 0.27 for RFs trained with all years—electronic supplementary material, figures S3 and S5). Improvements with RF trained for 2018 were particularly important for Eastern Europe, but low for Southern Europe (*R*^2^ < 50% for all crops).

### (b) Crop production anomalies in 2018 and their climatic determinants

**Figure 3.**
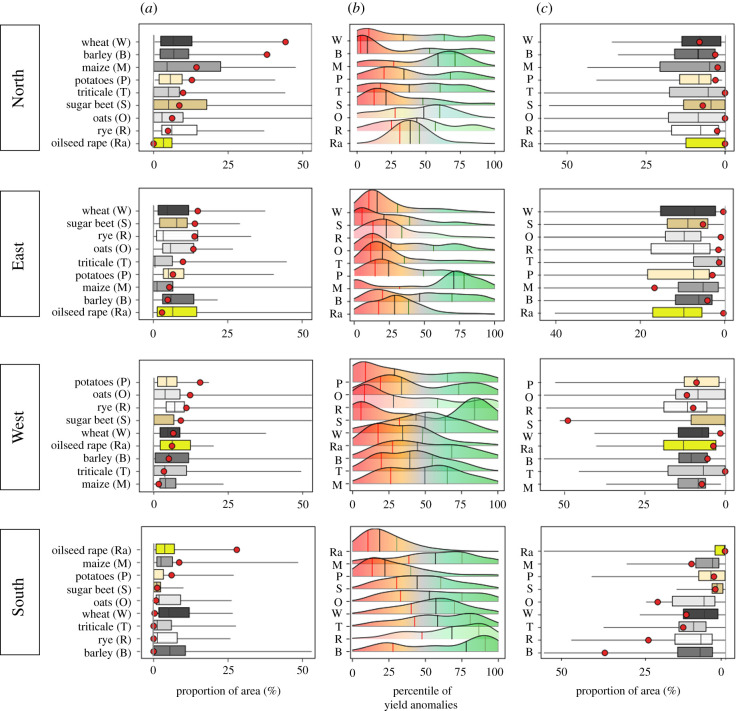
Yield anomalies and proportions of area with extreme yield loss and gain for nine crops in four European regions in 2018. Yield anomalies are characterized by their percentiles (*b*, percentiles in 2018), and proportions of area with extremely low (*a*) and high (*c*) yields in 2018 (red dots) compared to the whole time series (boxplots). Extreme yield losses and gains were defined as yields lower than the 10th percentile and higher than the 90th percentile, respectively. The crops are ordered within each region according to their proportion of extreme low yields. Red, black and green vertical lines in the density distribution of the crop yield anomalies correspond to percentile 25, 50 and 75%, respectively. Areas included in this plot are shown in figure 2. Names of the crops: T, triticale; M, maize, O, oats; P, potatoes; R, rye; Ra, oilseed rape; B, barley; S, sugar beet; W, winter wheat. (Online version in colour.)

**Figure 4.**
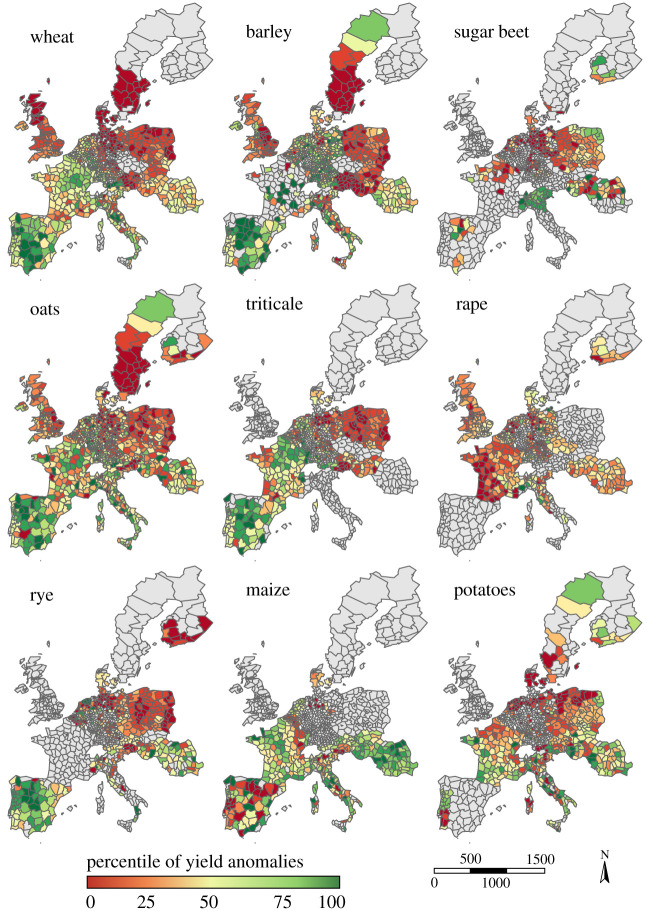
European yield anomalies in 2018 for the nine considered crops. Yield anomalies are expressed as the normalized percentile of the yield time series, and calculated as the difference between observed yields and expected yields estimated with loess regression. A percentile of 50% means that the observed yield corresponds to its expected value; a percentile of 10% (resp. 90%) means that these levels of low (resp. high) yields are observed, on average, once every 10 years. (Online version in colour.)

In 2018, Europe experienced multiple and simultaneous crop failures, Southern Europe excepted (figure 3). Median yield anomalies were in the lowest quartile of those observed since around 1990 for almost all crops, but yet varied greatly from region to region (figure 4).

**Figure 5.**
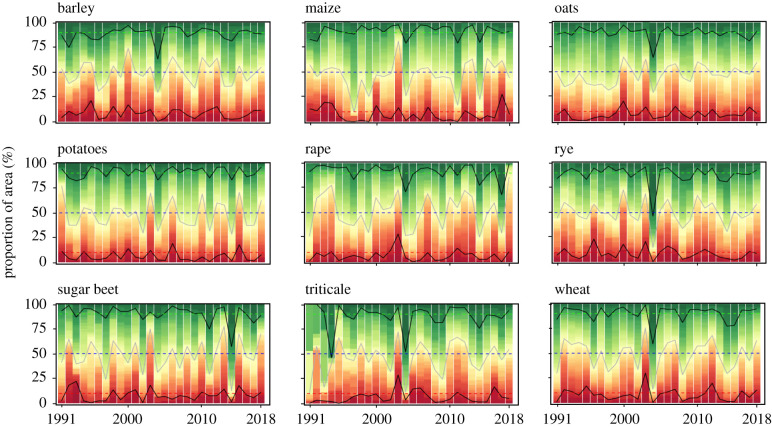
Proportions of cultivated area with positive (green) and negative (red) yield anomalies since 1990 in Europe for each of the nine crops. Colours represent the percentile of yield anomalies, with proportions of 10 and 90% highlighted with solid black curves, and of 50% with a solid grey curve. Dotted lines represent 10, 50 and 90% proportions of areas. The last year shown is 2018. (Online version in colour.)

**Figure 6.**
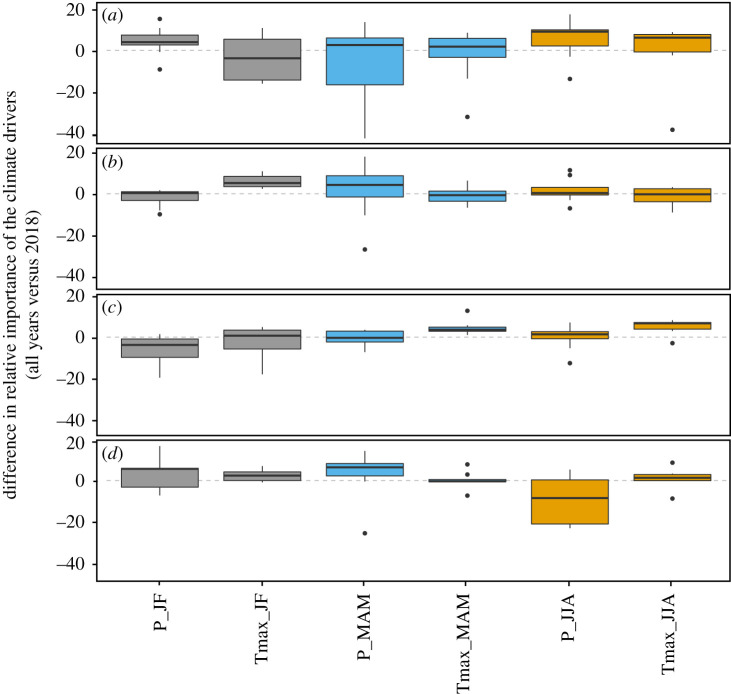
Variation in climatic driver relative (%) importance for random forest calibrated on long time series or only with year 2018 for four European regions: Northern (*a*), Eastern (*b*), Western (*c*) and Southern Europe (*d*). Boxplot represents the inter-crop variability. Colours corresponds to period of the crop cycle: grey, January–February; blue, March–April–May; orange, June–July–August. Relative importance is calculated based on a metric that captures the increase in mean squared error (MSE) of the random forest, calculated from out-of-sample predictions, after randomly permuting the values of the respective predictors. (Online version in colour.)

**Figure 7.**
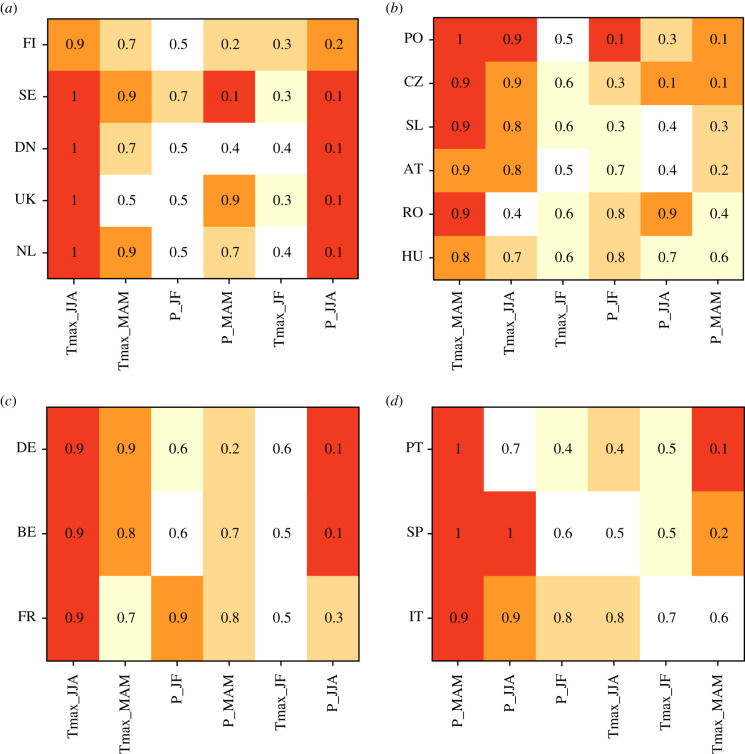
Percentiles of the climate anomalies in 2018 in various countries of four European sub-regions: (*a*) Northern, (*b*) Eastern, (*c*) Western and (*d*) Southern Europe. Only the six climate drivers that most impacted yields are shown (abscissa). Names of the countries (ordinates) are described in electronic supplementary material, figure S1. The climatic drivers are ranked by decreasing mean values (across countries) within each European sub-region. A percentile of 0.9 indicates that the value of the climatic variable is observed, on average, every 10 years. The higher (lower) the anomaly, the darker (lighter) the colour of the cell. (Online version in colour.)

Northern and Eastern Europe presented particularly negative anomalies in almost all districts, except for maize. Yield losses were particularly severe for winter wheat and barley with nearly 40% of Northern and Eastern Europe crop area recording yields below the 10th percentile (figure 4). In those two regions, nevertheless, a few positive yield anomalies were found, but only for a very small proportion of cultivated areas. Events of similar magnitude to 2018 had already been observed in the recent past, e.g. during the 2003 drought and heat wave (figure 5; electronic supplementary material, figure S9). A comparison of individual variable importance for RF either based on the full historical yield time series or only on 2018, revealed that some specific climate drivers played nonetheless a more important role in 2018 compared to all years (figure 6; electronic supplementary material, figure S6), but with some variations according to the region. In Northern Europe, high Tmax_MAM and Tmax_JJA values in combination with low summer rainfall (P_JJA) (figure 6) contributed substantially to explain yield anomalies in 2018. These climatic factors showed higher values than usually observed (figure 7), and impacted a large number of crop species (electronic supplementary material, figures S7 and S8). Other specific combinations of climatic conditions may also have worsened the situation in 2018, for example, below normal winter temperatures in Finland and Sweden (figure 1).

Eastern Europe experienced—sometimes simultaneously—various types of climate extremes (figure 7). The comparison of the importance of variables for RF models trained with 2018 data with those trained with historical data showed that Tmax_JF and P_MAM had a particularly strong influence on the 2018 yield anomalies in that region. The fact that these conditions are rare in the whole time series may explain the poor predictive quality of the RF calibrated on historical data to yields in 2018 events (electronic supplementary material, figures S3 and S5). P_MAM (along with P_JJA) showed values being among the lowest observed in recent years, while Tmax_MAM and Tmax_JJA had values above average (figure 7).

Western Europe experienced for most areas lower yields than expected for oilseed rape, rye, triticale and potatoes (figures 3 and 4). Triticale and maize showed large area with either extremely high or low yields. Positive and negative yield anomalies were also found for substantial proportions of barley and wheat growing areas, revealing that contrasting climatic conditions prevailed in different parts of Western Europe for these crops in 2018 (figures 3 and 4). For example, France experienced low wheat yields in the Southern and Western part of the country, but higher yields than expected in the Northern part. Overall, in Western Europe, yield in 2018 were not among the lowest observed since 1990. In terms of the extent of cultivated areas affected by negative anomalies, 2018 ranked fifth after 1992, 1998, 2007 and 2003 (figure 5; electronic supplementary material, figure S9). Western Europe showed larger impacts of temperature in the second part of the growing season (MAM and JJA) in 2018 compared to all years. Temperature showed higher values in the second part of the 2018 crop cycle than usually observed and were associated with lower rainfall (figure 7). Figure 1 shows that a compounding of climatic extremes worsens the individual effect of each variable.

Southern Europe experienced a high within-country variability of yield anomalies for several crops, particularly maize in Spain (figure 3). Only a small proportion of total cultivated areas in this region showed extreme yield losses, in particular for sugar-beet and rapeseed (figure 3), while more than 25% of the area in wheat, triticale, oats or rye showed highly positive yield anomalies (above the 90th percentile of distributions since about 1990; figures 3 and 4). Our results showed that in 2018, yields in Southern Europe benefited on average from wet spring conditions (P_MAM) with exceptional cumulated precipitation for all the countries in Southern Europe (figure 7).

Despite the fact that 2018 was characterized by extreme yields losses and gains, when investigating whether the frequency of extreme yield values (i.e. yield volatility) changed or not in recent decades, we found no clear upward or downward trend for any of the considered crops between 1990 and 2018 (figure 5). The years 2003, 2006, 2007 but also 1992, 1994 and 2000 showed a high proportion of cultivated area with extreme yield losses for various crops species (figure 5; electronic supplementary material, figure S10). Note that in 2010 there was extreme drought in summer over West Russia but our dataset do not cover this region. A similar result was found when aggregating all crops in each of the four regions separately (electronic supplementary material, figure S9).

## 4. Discussion

### (a) Extreme climate conditions for both dryness in Northern and Eastern Europe and wetness in Southern Europe

Only nine extreme summer conditions comparable with 2018 were identified from proxy-based seasonal paleoclimate reconstructions: five in the sixteenth century, three in the twentieth century, and one in 2003 [12]. Combining with spring climate anomalies, no contemporaneous similar event was reported in Eastern Europe. In 2018, Northern and Eastern Europe suffered from the coincidence of (i) dry spring conditions from late April, (ii) exceptionally high and persistent spring temperatures along with sunny conditions, and (iii) an abnormally dry and hot summer (figure 7; [12]). These climate extremes occurred during a key period of the growing season. Weather conditions in 2018 did become more favourable after mid-August, but these improvements were generally too small or occurred too late in crop cycles to significantly mitigate yield negative anomalies. This compounding of extreme conditions in 2018 led to one of the highest negative relative yield anomalies at the scale of Eastern and Northern Europe, across a large array of crop species (figure 3).

Heat episodes observed over the Northern Hemisphere in 2018 were likely amplified by human-induced climate change [8,9,31]*.* Climate change has increased the frequency (how often events occur), intensity (how high a temperature/how dry a drought) and the duration (how long they last) of extreme events [2]. Future climate projections reveal that these events could become the norm as early as approximately 2050 in central and Northern Europe [12,32,33]*.* Increased variability of climate, and occurrence of more frequent extreme climatic events, e.g. drought events, could offset or increase estimated mean impacts of climate change on agricultural production [34,35]. Climate change is projected to contribute to a longer growing season in Nordic countries, possibly resulting to increased crop yields [36,37]. Our study shows that large scale yield losses are not to be excluded in that region in the event of severe drought, that could strongly impact the long-term productive and economic efficiency of agriculture. Considerable uncertainty and knowledge gaps remain to assess the impacts and adaptation of Nordic and Eastern agriculture to climate change [38].

Our analysis also points out that 2018 was a contrasted year at the European scale, because Southern Europe experienced positive anomalies for the majority of the crop species considered in this study (figure 3). We demonstrated that these high yields are partially explained by favourable spring conditions, with one of the two wettest springs since 1950 in Southern Europe. Wet conditions in the spring were combined with one of the wettest summers in the last seventy years (figure 7; [20]). These anomalous weather conditions—notably in March—were linked to a persistent negative North Atlantic Oscillation pattern (NAO; [39]).

Higher yields in Southern Europe compensated for the massive production loss in Eastern and Northern Europe. As a result, Europe-wide cereal production dropped only by 8% compared to the 5-year average [40]. Relying on a complete production compensation through market forces between European countries or at global scale may not be a viable climate change adaptation option. Climate extremes in a key producing country can induce global price spikes and modify trade patterns with effects going beyond the year of occurrence [41], and self-propagating trade disruptions [42]. For example, the lower 2018 production in Europe resulted in spiking cereal prices with an extra €50 per ton for wheat (base: €170 in May 2018) and an added €60 per ton for barley (August prices [40]). Extreme climatic episodes of 2018 were also associated with above-normal temperatures in North America and the Caspian Sea region [43]. These global climate modes influence a substantial proportion of crop production variance, e.g. approximately 14% of winter wheat in Europe for the NAO [44]. The probability of synchronous crop yield anomalies in various regions of the world would increase with climate change, e.g. 26% and 28% higher risks, respectively, for maize and wheat for a global warming of 1.5 °C compared to 2°C [3].

### (b) Climate variables explain a substantial part of the yield variability in Europe

The set of uni-scalar climate variables included in the RF models calibrated on historical yield data explained 34% of the variability of yield anomalies on average, up to 65% in some crops and European regions (electronic supplementary material, figure S3). The proportion of explained variance was lower in some specific combinations of crops and regions. Previous studies modelling national or sub-national yields based on climatic variables reported a similar level of explained variance. Lobell & Field [45] explained about 30% of year-to-year variations at the global scale (results without cross-validation), similarly Ray *et al*. [46] showed that about one-third of the variability in yields was explained by climate variation worldwide (results obtained without cross-validation). Using mean values and extremes events, Vogel *et al*. [8,9] explained up to 50% of the variability for various crop at continental scale (based on an out-of-bag cross validation). Based on climatic variables but also soil properties and management, the machine learning (random forest, XGBoost) algorithm used by Shahhosseini *et al.* [47] explained between 35 and 56% of variation of maize yields simulated by a crop model in the US. In our study, the unexplained part of yield variability may be due to a number of non-climatic factors, such as crop management (e.g. availability and use of inputs, soil management), pest and diseases, political and social context (e.g. [8,9,46]). The lower fraction of the yield anomalies variance explained for Southern European countries (mean *R*^2^ = 15%) in our study may be partly due to a lower or more heterogeneous level of data quality. Note the non-distinction between irrigated and rain-fed crops in our data from available statistics despite the fact that most of the European irrigated areas are located in Southern Europe [48]. The shorter yield time series and the lower number of districts included in our study for Southern Europe (only 10% of the total number of yields data in our database) may also explain the reduced RF predictive ability in this region (electronic supplementary material, figure S1). More generally, despite the efforts in the various European countries to use homogeneous methods and provide high-quality data, a certain degree of subjectivity is associated with these regional statistics, and could thus increase the amount of unexplained yield variation.

Global improvements of the predictive quality of our models are possibly reachable using other climatic variables, or other temporal aggregations. More complex climate inputs are sometimes used in yield forecasting studies. Drought indices, e.g. the Standardized Precipitation Evapotranspiration Index, have sometimes been shown to perform better (e.g. [49]) than simpler indices. Yet, there is no consensus on a positive relationship between the level of complexity of an indicator and their accuracy, e.g. to predict extreme wheat and maize yield losses [21] or for drought [22]. Similarly, some studies suggested that the use of higher temporal resolution (i.e. at monthly or infra-monthly climate inputs, or depending precisely on the crop cycle) could improve the predictive quality of the models in such types of analyses [50,51]. Yet, crops reach the physiological stage at which they are sensitive, for example, to temperature stress, at different times of the year, which depend on the crops, geographical areas and sowing dates, and are sometimes difficult to estimate precisely on a large scale. Furthermore, Ben-Ari *et al.* [21] did not find any added value in considering climate aggregation based on precise estimation of the crop phase (i.e. vegetative and reproductive) compared to monthly, bi- or tri-monthly aggregation. Similarly, Sharif *et al.* [52] did not find any advantages to considering fortnightly rather than monthly aggregated climate variables to predict yields. We estimated the impatcs of climate on yield anomalies in four European regions (based on pairs of climate and yield data for each district and year available). Local geographical disparities could have hampered a precise estimation of the parameters of the RF. The relatively low share of explained variation in Eastern Europe (mean *R*^2^ = 34%, across all crops) could also stem from local specific climatic conditions. Hungary, with its Pannonian climate, could experience significantly different climatic conditions than its neighbouring countries (e.g. in 2018, figure 7).

RF models calibrated from all years suitably predicted 2018 yield anomalies in Northern and Western Europe (electronic supplementary material, figure S3 and figure S5), suggesting that this combination of key climate events had already been observed—even at lower intensity. The unforeseen compound climate event observed in Eastern Europe in 2018 has probably impaired the ability of models to correctly estimate yield anomalies in 2018. The estimated effects of main climatic drivers on yield largely changed in this part of Europe for RF calibrated on historical time series (figure 6) or only on 2018 (electronic supplementary material, figure S11).

### (c) Low yields are often caused by climatic anomalies, in single and compound actions

We identified the most influential climate drivers (and their thresholds) impacting yields over time over Europe. Among the tested climate variables, we found that temperature- and precipitation-related predictors have higher importance than soil moisture (0–7 cm). In each region, most of the estimated climate driver effects on yield anomalies were robust across the same types of crop (figure 2; electronic supplementary material, figure S7 and S8). Effects were also robust to the type of machine learning algorithm and parameter tuning and the type of yield time series detrending methods (electronic supplementary material, figure S2).

High maximum temperature (24°C) had a particularly negative impact on yields in Eastern and Western Europe (figure 2). The strong association between temperature, particularly high temperatures, and yields is consistent with previous research at the national [1,53] or global [8,9,54] scales. The impact of high temperatures on production is caused by individual or a combination of above average temperature for an extended period, and heat shock characterized by very high maximum temperature. These stresses could reduce flower fertility, limit grain number and weight, for example by limiting the duration of the grain filing period [55]. Critical thresholds of temperature depend on phrenological stages and are sometimes inconsistent between studies (e.g. number of days with a temperature over 25°C: [56,57]; 30°C: [50]). Instead of using a fixed threshold of temperature, Ben-Ari *et al.* [21] established a continuous relationship between temperature and the probability of extreme yield losses. In Northern Europe, no negative impacts of high temperature were observed, possibly because maximum temperature values averaged over JJA rarely exceed 25°C. In Southern Europe, high temperature did not negatively impact yields (figure 2). The use of tolerant cultivars could explain this high crop performance under heat [58].

Frost (T° < 0°C) showed large impacts in Western Europe and Eastern Europe (figure 2). Frost could affect seedling survival, and cause leaf or bud damage by the formation of ice crystals in plant tissues [55]. Impacts in Northern Europe could be limited by the use of tolerant cultivars (some genotypes are tolerant to a temperature of −20°C, [59]) and cold acclimation [60].

We also found that rainfall scarcity (less than 2.5 mm day^−1^ in MAM for Eastern, Western and Southern Europe) or excess (greater than 4 mm day^−1^ in Northern, Eastern and Western Europe) largely negatively impacted crop yields over the full period considered (figure 2). Abnormally low rainfall along with high temperatures can increase drought severity and are often significantly correlated [61,62]. The negative impact of the co-occurrence of such stresses have already been highlighted for cereals crops (e.g. [51,63] for barley crops in Western Europe). On the contrary, the impacts of excessive rainfall on crop production, as shown by our analysis of historical data (figure 2) in Eastern and Northern Europe, seem to be far less studied [64]*.* High rainfall could reduce production through, for example, damage from oxygen deficit as a consequence of soil waterlogging after heavy rain [65,66]; bending of the stem [67]; or erosion, loss of soil nutriment and plant anchorage failure. Li *et al*. [68] and Huang *et al.* [69] demonstrated that excessive rainfall can adversely affect maize yields in the USA in proportions similar to extreme drought. These impacts may become more frequent in the future given the expected increase in the frequency of extreme precipitation events [70].

Finally, our study showed that yield anomalies are also explained by compounds of climate variables occurring throughout crop cycles, e.g. temperature in January–February and rainfall in March–April–May for wheat in most of the European regions (figure 7). This inter-dependence of climatic factors to explain yield losses have already been highlighted in various studies (e.g. [51] on historical data for barley in France, [10] for wheat in 2016 in France; [71] for maize at a global scale). Variation in climate modes can partly explain the co-occurrence of climate variables unfavourable to yields [72]. For example, comparing recent major droughts in Europe, similar preceding rainfall deficits and strong feedbacks between air temperature and soil water anomalies were observed preceding the 2003, 2010 and 2018 droughts [73–76]. Yet, the area under droughts and factors aggravating the effect of the drought are distinct: severe soil drying caused by preceding rainfall deficits and high evaporative demand prior to summer in 2003, and high evapotranspiration linked to extreme warm and sunny conditions in spring in 2018 [76]. The resulting impacts on ecosystems differed for these three events (figure 5; electronic supplementary material, figures S9 and S10; [76]). The proportions of areas with very low yields were higher in 2003 compared to 2018, notably for triticale, oilseed rape, sugar beet and wheat (electronic supplementary material, figure S9, S10), yet both droughts seemed to have impacted particularly Northern and Eastern Europe (all crops included; figure 5). As in 2018, both high temperature and low rainfall seemed to be responsible for the yield losses [77].

Globally, regarding the impacts of the 2018 specific climate conditions, the key findings of our study are that: (i) climate variables explained a large part of yield anomalies in that year (i.e. mean *R*^2^ = 50%), (ii) the relative importance of climatic variables were different from those usually observed in the four European regions, (iii) these variables differed across regions and (iv) corresponded to extreme values. Northern European crop yields have been more strongly impacted by JJA conditions, Eastern and Southern European yields by MAM precipitation patterns and Western crop yields by maximum temperature over the spring and summer (MAM and JJA).

## 5. Conclusion

In 2018, Northern and Eastern Europe experienced above normal yield losses which can be explained by compounds of climate extremes occurring at different periods of the crop growing cycle. These regions suffered from a major heatwave in spring and summer. The higher than usual yields recorded in Southern Europe—caused by favourably wet spring conditions—did offset these losses hence preventing a large decrease in crop production at the European scale. Our results show that, in most situations, simple climate variables can explain a large fraction of the variability of yield anomalies, with a few exceptions, especially in Southern Europe. Our results outline the importance of considering regionally specific single and compound climate extremes to analyse the causes of yield loss in Europe.

## Supplementary Material

Additional tables and figures

## Data accessibility

Our study is based on public data. We have not generated any new data: all climate data are publicly available (https://www.ecmwf.int/en/forecasts/datasets/reanalysisdatasets/era5) and can be easily accessed. All yield data arise from publicly available data and are easily accessible from the various national statistical offices of the European countries.
