# Supplementary material for: Impact of extreme weather conditions on European crop production in 2018
Source: Philos Trans R Soc Lond B Biol Sci. 2020 Sep 7;375(1810):20190510. doi: 10.1098/rstb.2019.0510 (PMC7485097; doi:10.1098/rstb.2019.0510)
Supplement: Additional tables and figures [file rstb20190510supp1.docx]

**Supplementary materials:**

Supplementary materials to the article “Beillouin D, Shauberger B, Bastos A, Ciais P, Makoswki D. 2020 Impact of extreme wheather conditions on the European crop production in 2018. *Phil. Trans. R. Soc. B.* 20190510”

**Figure S1.** Duration of yield time series for the 17 European countries and the nine studied crops in the four European regions: Northern Europe (A), Eastern Europe (B.), Western Europe (C.) and Southern Europe (D.). The colors and the number represent the number of years of available data for each combination of crop and country. The length of yields time series could slightly differ within each country, only the maximum duration is mentioned in this plot.

AT: Austria, BE: Belgium, DN: Denmark, FI: Finland; FR: France, DE: Germany, HU: Hungary, IT: Italy, NL: The Netherlands, PO: Poland, PT: Portugal, RO: Romania, CZ: Czech Republic; SL: Slovakia, SP: Spain, SE: Sweden, UK: United Kingdom.

**Figure S2.** Sensitivity analysis of the estimated impacts of climate drivers on normalized yield anomalies for four variables P_JJA (A.), Tmax_JJA (B.), TmaxMAM (C.) and P_MAM (D.). The sensitivity analysis was run on Winter wheat for Northern European region. Color of the curve correspond to type of detrending method of the yield time series (blue: loess detrending; red: polynomial detrending; green: spline detrending). Line types correspond to methods for estimating impacts of climate factors on normalized yields anomalies (solid: pdp –partial dependence plot, dotted: ale- accumulated local effects plots). We also tested two machine learning algorithms: random forest and gradient boosting (not differentiated on the plot), both with two set of tuning parameters.

**Figure S3.** Explained variance of normalized yield anomalies (R^2^) of various random forest (RF) models per crop and European regions: Northern Europe (A), Eastern Europe (B.), Western Europe (C.) and Southern Europe (D.). Gray bars correspond to R^2^ (cross-validation with a validation set of 25% of the data) for all years for RF calibrated on long time-series. Orange bars correspond to R^2^ of year 2018 for random forest calibrated on long time-series. Blue bars correspond to R^2^ (cross-validation with a validation set of 25% of the data) of years 2018 for RF calibrated only with year 2018.

**Figure *S*4.** Relative importance of the variables explaining yield anomalies in four regions of Europe and the nine crops, based on a random forest calibrated with all years. The variable importance corresponds to the impurity measured by the Gini index calculated in the random forest. The relative importance is then calculated as the proportion of importance of each variable over the sum of importance. Higher values correspond to a stronger influence of the respective variable onto yield anomalies. The type and colors of the curve indicate the four regions of Europe. The x-axis is ranked by averaged mean importance over all nine crops.

**Figure S5.** Comparison of the proportion of variance of normalized yield anomalies (R^2^) explained for year 2018 based either on a random forest calibrated with historical-time series (x-axis) or only with year 2018 (y-axis). The colors correspond to the European regions: Northern Europe (green), Eastern Europe (red), Western Europe (violet) and Southern Europe (blue).

**Figure S6.** Relative importance of the variables explaining yield anomalies in four regions of Europe and for the nine crops, based on a random forest calibrated only with 2018. The variable importance corresponds to the impurity measured by the Gini index calculated in the random forest. The relative importance is then calculated as the proportion of importance of each variable over the sum of importance. Higher values correspond to a stronger influence of the respective variable onto yield anomalies. The type and colors of the curve indicate the four regions of Europe. The x-axis is ranked by averaged mean importance over all nine crops.

**Figure S7.** Estimated impacts of main climate drivers on maize and sugar-beet yield anomalies in four European regions: Northern Europe (A.), Eastern Europe (B.), Western Europe (C.) and Southern Europe (D.). Gray curves correspond to the effects of each crops estimated independently by random forests on normalized yields anomalies. Only the six main drivers are presented in this plot. Blue curves correspond to a loess fit. Blue segments correspond to the experienced value of climate drivers in 2018 for countries within each region.

**Figure S8.** Estimated impacts of main climate drivers on barley spring (red) and rape (blue) yield anomalies in four European regions: Northern Europe (A.), Eastern Europe (B.), Western Europe (C.) and Southern Europe (D.). Green and red curves correspond to the effects of each crops estimated independently by random forests on normalized yields anomalies. Only the six main drivers are presented in this plot. Blue segments correspond to the experienced value of climate drivers in 2018 for countries within each region.

**Figure S9.**  Years with highest proportion of area with high normalized yield anomalies per crop over Europe since 1990. 2018 is highlighted in blue.

**Figure *S10*.** Proportion of area with various yields anomalies since 1990 in four regions of Europe: Northern Europe (A.), Eastern Europe (B.), Western Europe (C.) and Southern Europe (D.). Anomalies are calculated as the mean of the nine considered crops. Colors represent the percentile of yields anomalies, with percentile 10 and 90% highlighted with a black curve, and percentile 50% with a gray curve. The last year corresponds to 2018.

**Figure S11.** Normalized yields anomalies estimated for the climate values observed in 2018 with random forests calibrated for wheat and only in 2018 or all years in four European regions: Northern Europe (A.), Eastern Europe (B.), Western Europe (C.) and Southern Europe (D.). Only the six most importance climatic drivers are presented. The colors correspond to the phase of the crop cycle: gray: January-February (JF), blue: March-April-May (MAM) and orange: June-July-August (JJA).
